# Pneumothorax and Pneumomediastinum in Pregnancy: A Case Report

**DOI:** 10.1155/2009/465180

**Published:** 2009-12-29

**Authors:** S. Sathiyathasan, K. Jeyanthan, G. Furtado, R. Hamid

**Affiliations:** Mayday University Hospital, Croydon, Surrey CR77YE, UK

## Abstract

*Case Report*. A 37 years old patient at 40 weeks gestation presented with acute severe hypoxia with a seizure followed by fetal bradycardia. Caesarean section was performed under GA and she was intubated and ventilated. History revealed longstanding right pleural endometriosis with multiple pneumothoraces and hydrothoraces. A CT chest showed extensive bilateral pnenumothoraces. Her clinical condition improved with a left-sided chest drain. *Discussion*. Severe hypoxia and seizures in a patient with previous history of pnenumothorax are highly suggestive of tension pneumothorax. Radiological investigations are vital for diagnosis. The traditional treatment approach to recurrent pneumothorax has been thorocotomy with bleb or bulla resection and pleurodeisis. The advantages of thorocoscopic surgical treatment over thorocotomy are decreased time of exposure to anaesthetic drugs, rapid lung expansion, decreased post operative pain, and a potentially shorter post operative recovery. In any future pregnancy due to the high risk of recurrence of pneumothorax Contemporary obstetric management should determine the method of delivery and continuous lumbar/epidural anesthesia should be used if at all feasible. Preconceptual counseling about this risk is vital, and women must be advised about potential serious adverse outcomes.

## 1. Introduction

Spontaneous acute pneumothorax during pregnancy is extremely rare and potentially serious for both the patient and fetus [1, 2]. We present a case of pneumomediastinum and pneumothorax in a 37-year-old primiparous woman and then discuss the clinical presentation, management, and prognosis.

## 2. Case Presentation

A 37-years-old primigravida at 40 weeks gestation was brought to our hospital by ambulance following collapse at home. No obstetric notes were brought in. On admission she was anxious, apyrexial, dyspnoeic, and distressed.

**Figure 1:**
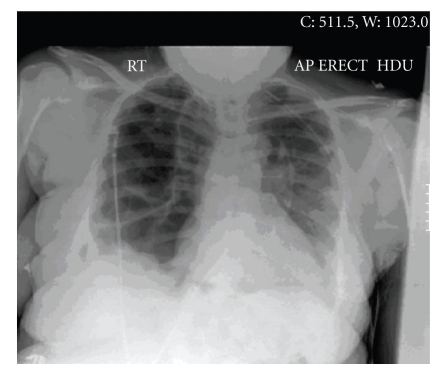
Pnemothorax postchest drains insertion, new left chest drain in situ. There has been good re-expansion of the left lung. No definite right pneumothorax seen.

**Figure 2:**
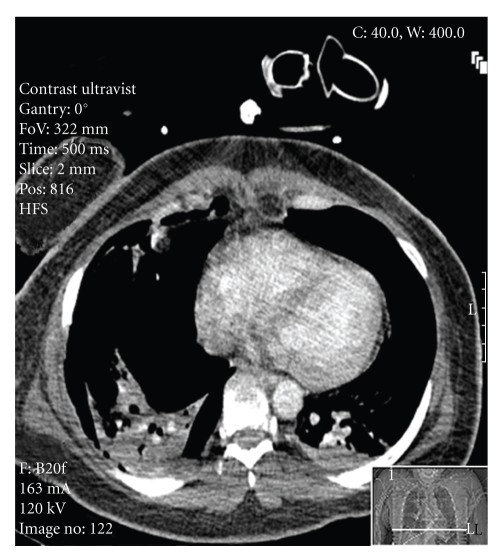
Extensive bilateral pneumothoraces.

On clinical examination she was hypoxic, with oxygen saturation of 68% on air and respiratory rate of 30/minutes. Blood pressure was 150/90, and Glascow coma scale was 11/15. There were no signs of labour. Soon after arrival she had a seizure. Following the seizure there was an episode of fetal bradycardia and an urgent caesarean section was performed under GA. The baby was delivered in good condition. There was no evidence of placental abruption or any other cause for fetal bardycardia. Patient's vitals were stable during the caesarean section. She was transferred to Intensive Care Unit, where she was intubated and ventilated. A CT chest showed extensive bilateral pnenumothoarces of greater than 70% in the left side and extensive opacities in the semicollapsed right lower lobe (see Figures 1 and 2). The CT head was normal. A left-sided chest drain was inserted on the same day. This was removed on the following day after patient's clinical condition had improved. Patient has no symptoms or signs which suggestive of Pre-eclamsia.

Following the caesarean section the family arrived. At this stage we found that the patient had longstanding right pleural endometriosis with multiple pneumothoraces and hydrothoraces and underwent right-sided pleurodesis in 2003. This lady was booked in at tertiary hospital for pregnancy care. She had regular followups with a Chest physician and Obstetrician. At 29 weeks gestation the patient had presented with mild dyspneoa and the chest X-ray revealed a small effusion but no significant pneumothorax. The cardiothoracic surgeon suggested that since the pleura was well stuck down, there was a low risk for expanding pnuemothorax. This patient still presented with severe hypoxia with pneumothorax. The Cardiothoracic surgeon further advised assistance with instrumental delivery as small risk of increased pneumothorax in the second stage of labour during pushing. Epidural or spinal anaesthesia was recommended and to avoid General anaesthesia. If general anaesthesia was needed then intermittent positive pressure ventilation should be used. The Obstetrician had planned for induction of labour at 41 weeks gestation. On the 4th day of postcaesarian section, the patient again complained of shortness of breath. On general examination the patient was dyspneic and tachypneic. Her blood pressure was normal, she had no proteinuria, and her liver function and platelets were within normal limits.

Chest X-ray showed that she had left-sided lower lobe pneumonia and was started on intravenous tazocin, IV gentamicin, and then on oral erythromycin. Patient recovered well and was transferred to a tertiary hospital.

## 3. Discussion

This patient presented with dyspnoea and severe hypoxia at a district general hospital without any clinical notes and was brought by ambulance. No relatives accompanied her which made the clinical management difficult. She needed intubation and ventilation as an emergency measure to manage her severe hypoxia.

In pregnancy there is a 20% increased oxygen demand and during labour this is 50%. Consequently any impairment of ventilation in pregnant women may lead to hypoxia more readily than nonpregnant women [3]. During pregnancy, minute ventilation, tidal volume, and respiratory rate are increased. Despite a 20% increase in oxygen consumption and a 70% increase in alveolar ventilation, the functional residual capacity is decreased from 17% to 20%. Thus any additional stress upon the maternal respiratory system represents a serious potential hazard to fetus. Further, the fetal umbilical vein Po2 is only 35 to 45 mm hg and any decrease in maternal Oxygen saturation is potentially life threatening to the developing fetus [4]. As long as fetal oxygen reserves are not depleted, fetal metabolic functions will continue aerobically, even though fetal hypoxemia is present. As O2 reserves are exhausted in some tissues, fetal hypoxemia will be associated with tissue hypoxia, the net result of which will be anaerobic metabolism, lactic acidosis, and tissue death [5]. The most common preceding complaint is a history of a previous pneumothorax [6]. This patient had multiple pnenumotoraces in the past and a small residual hydropneumothorax diagnosed antenatally. Clinically the condition may present with substernal chest pain, dyspnoea, dysphonea rarely dysphagia. Surgical emphysema of neck and face is pathognomonic [7]. The Electrocardiogram is not very useful but changes may include sinus tachycardia with ST and T wave abnormalities in 25% of sufferers [7, 8]. The chest X-ray is diagnostic [7, 9, 10]. The differential diagnosis includes cardiac tamponade, pericarditis, angina pectoris, dissecting aneurysm, mediastinitis, pulmonary embolism and oesophageal tear [7].

Maternal collapse with severe hypoxia and seizures in a patient with previous history of pnenumothorax is highly suggestive of tension pneumothorax. Other differential dignosis is massive pulmonary embolism, eclampsia, cerebral haemorrhage, and epilepsy. Most patients with spontaneous pneumothorax require only conservative management consisting of reassurance, oxygen supplement, and analgesics [11]. Admission and close observation of the patient is usually done with small pneumothoraces. Other treatment options are needle aspiration, needle decompression, pleurodesis, tube thoracostomy, and thoracoscopy for recurrent, persistent or bilateral pnenumothorax [1]. Treatment options are generally the same as for nonpregnant patients; however any ventilatory problems associated with pneumothorax may not be well tolerated by a pregnant patient and her fetus [12]. 

Surgical management of spontaneous pneumothorax during pregnancy is well recognised. Indication for surgical treatment is persistent, or multiple recurrent pnemothoraces [13]. Nonetheless surgery should be avoided if possible [14]. Indications for operative treatment of pnumothorax in general are not absolutely defined. The traditional treatment approach to recurrent pneumothorax has been throcotomy with bleb or bulla resection and pleurodesis. Recently thorocoscopy is more frequently used. The advantages of throcoscopic surgical treatment over thorocotomy are decreased time of exposure to anaesthetic drugs, rapid lung expansion, decreased postoperative pain, and potentially more brief postoperative recovery [12]. 

Thorocotomy incision and resultant pain severely limit mother's mobility and result in impaired bonding. There is a reported increase in incidence of postpartum depression in such cases [6]. In this case caesarean section also was justified for an obstetric reason of fetal bradycadia. Normally caesarean sections are only performed for Obstetric reasons in patients with pneumomediatinum or pneumothorax [6].

In labour, the use of epidural is recommended in patients with pneumothorax as it avoids futher exertion and provides good analgesic that can easily be converted to anesthesia for forceps or caesarean delivery. A subarachnoid block should be administered cautiously if required as high spinal block may compromise respiratory function. If general anesthesia is indicated, facilities for chest drain should be immediately available and nitrous oxide must be avoided [9]. If the initial pneumothorax is large (more than 15%–20% of hemi thorax) aspiration is recommended. This patient presented with severe hypoxia, respiratory arrest, and a seizure therefore she needed intubation and this could have resulted in further expansion of the pneumothorax [14]. Pneumothorax should be considered in any pregnancy women with chest pain and dyspnoea and must be confirmed radiographically to distinguish it from other conditions. Though this patient had a history of multiple pneumothoraces and hydrothoraces, diagnoses were difficult as she presented as an emergency. Her previous history was unknown to medical staff.

In any future pregnancy due to high risk of recurrence of pneumothorax one should strongly consider delivery when fetal pulmonary maturity can be documented. Contemporary obstetric management should determine the method of delivery, continuous, lumbar; epidural anesthesia should be used if at all feasible [6].

Studies describe an increased risk of recurrence both in pregnancy and during childbirth in patients with pneumothorax. Patients with a history of pneumothorax who are contemplating pregnancy need to be aware of the risk of recurrence during pregnancy, parturition, or shortly after normal delivery or cesarean section [15]. Preconceptional counselling about this risk is vital and women must be advised about potential serious adverse outcomes.

## References

[1] Gorospe L, Puente S, Madrid C, Novo S, Gil-Alonso JL, Guntiñas A (2002). Spontaneous pneumothorax during pregnancy. *Southern Medical Journal*.

[2] Johnson SR, Tattersfield AE (2000). Clinical experience of lymphangioleiomyomatosis in the UK. *Thorax*.

[3] Van Winter JT, Nichols FC, Pairolero PC, Ney JA, Ogburn PL (1996). Management of spontaneous pneumothorax during pregnancy: case report and review of the literature. *Mayo Clinic Proceedings*.

[4] Leontic EA (1977). Respiratory disease in pregnancy. *Medical Clinics of North America*.

[5] Edelstone DI (1984). Fetal compensatory responses to reduced oxygen delivery. *Seminars in Perinatology*.

[6] Farrell SJ (1983). Spontaneous pneumothorax in pregnancy: a case report and review of the literature. *Obstetrics and Gynecology*.

[7] Gemer O, Popescu M, Lebowits O, Segal S (1994). Pneumomediastinum in labor. *Archives of Gynecology and Obstetrics*.

[8] Sutherland FWH, Ho SYG, Campanella C (2002). Pneumomediastinum during spontaneous vaginal delivery. *Annals of Thoracic Surgery*.

[9] Hague WM (1980). Mediastinal and subcutaneous emphysema in a pregnant patient with asthma. Case report. *British Journal of Obstetrics and Gynaecology*.

[10] Karson EM, Saltzman D, Davis MR (1984). Pneumomediastinum in pregnancy: two case reports and a review of the literature, pathophysiology, and management. *Obstetrics and Gynecology*.

[11] Miguil M, Chekairi A (2004). Pneumomediastinum and pneumothorax associated with labour. *International Journal of Obstetric Anesthesia*.

[12] Galle PC, Servoss RL, Warren TL (1979). Spontaneous pneumothorax occurring during labor and delivery. *American Journal of Diagnostic Gynecology and Obstetrics*.

[13] Dhalla SS, Teskey JM (1985). Surgical management of recurrent spontaneous pneumothorax during pregnancy. *Chest*.

[14] Wong MK, Leung WC, Wang JK (2006). Recurrent pneumothorax in pregnancy: what should we do after placing an intercostal drain. *Hong Kong Medical Journal*.

[15] Johnson SR, Tattersfield AE (2000). Clinical experience of lymphangioleiomyomatosis in the UK. *Thorax*.

